# A Comparison of Measures for Assessing Profile Similarity in Dyads

**DOI:** 10.5334/pb.1297

**Published:** 2024-06-25

**Authors:** Chiara Carlier, Julian D. Karch, Peter Kuppens, Eva Ceulemans

**Affiliations:** 1 Department of Psychology and Educational Sciences, KU Leuven, Belgium; 2 Department of Methodology and Statistics, Institute of Psychology, Leiden University, The Netherlands

**Keywords:** Similarity, distance, measures, profiles, dyads, clustering

## Abstract

Profile similarity measures are used to quantify the similarity of two sets of ratings on multiple variables. Yet, it remains unclear how different measures are distinct or overlap and what type of information they precisely convey, making it unclear what measures are best applied under varying circumstances. With this study, we aim to provide clarity with respect to how existing measures interrelate and provide recommendations for their use by comparing a wide range of profile similarity measures. We have taken four steps. First, we reviewed 88 similarity measures by applying them to multiple cross-sectional and intensive longitudinal data sets on emotional experience and retained 43 useful profile similarity measures after eliminating duplicates, complements, or measures that were unsuitable for the intended purpose. Second, we have clustered these 43 measures into similarly behaving groups, and found three general clusters: one cluster with difference measures, one cluster with product measures that could be split into four more nuanced groups and one miscellaneous cluster that could be split into two more nuanced groups. Third, we have interpreted what unifies these groups and their subgroups and what information they convey based on theory and formulas. Last, based on our findings, we discuss recommendations with respect to the choice of measure, propose to avoid using the Pearson correlation, and suggest to center profile items when stereotypical patterns threaten to confound the computation of similarity.

## Introduction

For over half a century many behavioral studies examined questions that pertain to the similarity of multivariate profiles. Such a profile is formed by measuring multiple variables for a person at a given measurement occasion. A profile can then be similar or dissimilar to another profile. A few examples of profile similarity focused research questions are whether immigrants show similar emotional patterns as members of their host culture, whether sons exhibit similar personality traits as their fathers and how the emotional similarity of two romantic partners fluctuates when they go about their daily lives (see e.g., De Leersnyder et al., 2011; McCrae, 2008; Sels, Ruan, et al., 2020). These few examples showcase the diversity of profile similarity research. In the first and last case, the set of variables pertains to several discrete emotions, and in the second case, to the Big Five personality traits. Additionally, in the last two cases, similarity is computed between two people, whereas in the first case, it is computed between an individual and a cultural norm obtained by averaging the profiles of multiple people. Finally, whereas the second case can be assessed in a cross-sectional way, the third case requires an intensive longitudinal design in which each partner is measured multiple times. In this paper, the focus will lie on longitudinal and cross-sectional sets of emotion profiles obtained from both individuals and cultures.

Once data have been collected, researchers have to decide how they will quantify profile similarity. In the literature, the Pearson correlation or the Euclidean distance are often used (e.g., Allik et al., 2015; De Leersnyder et al., 2013; Sels, Ruan, et al., 2020; Terracciano et al., 2005). However, the recurring choice for the Pearson correlation or the Euclidean distance is often not well motivated in terms of what type of information researchers aim to extract from these measures. This is worrisome since it is known that the choice of measure can impact conclusions (Furr, 2008; Kenny et al., 2006). Moreover, several other profile similarity measures have been developed. For instance, Cattell (1949), Cohen (1969), and McCrae (1993) have all developed measures specifically tailored to comparing profiles (Cattell, 1949; Cohen, 1969; McCrae, 1993). In addition, outside the field of profile similarity, many additional measures have been proposed that are potentially useful. Székely et al. (2007), for instance, developed a distance correlation coefficient that is only zero when there is no association between the variables at all (Székely et al., 2007). In linguistics literature, Burrows’s Delta (Burrows, 2002) has been used to assess how similar two manuscripts are, and in ecology, the Morisita-Horn index (Krebs, 2014) can tell how much two species overlap. Looking at still other fields, yield even more possibilities (Deza & Deza, 2009).

In light of this wealth of available measures to study profile similarity, for researchers to choose the similarity measure that appropriately formalizes their research question, it is important to have precise information on how all these measures are related and what type of information they convey. To this end, in this paper, we aim to scrutinize a wide range of possible profile similarity measures, clarify their similarities and differences and provide recommendations for their use. Specifically, we have compiled a list of similarity measures by looking into software packages and reviewing papers. We have installed some criteria to only include measures for which analytical formulas are available, including two vectors. Starting from this list, we sequentially addressed four questions. First, which similarity measures are applicable to measure profile similarity and yield unique values of profile similarity? Some measures might not be suited for analyzing profiles based on ratings or scores. Additionally, there might be some measures that go by different names but give duplicate or complementary values. They can then be reduced to one measure.

Second, can the subset of applicable measures be subdivided into similarly behaving groups? Classifying a long list of similarity measures into similarly behaving groups can help to guide choices. Cronbach and Gleser (1953) and also later authors, made an all-encompassing distinction between Euclidean distance-like and correlation-like measures based on their formulas: whereas distance measures are based on difference scores, correlation measures are based on product scores (Skinner, 1978). Kenny et al. (2006) largely focused on the distinction between similarity and dissimilarity measures, which is, however, closely related to the difference versus product distinction (Kenny et al., 2006). The papers discussed only considered profile similarity measures commonly used in psychological research. Given that we also consider measures that were proposed in other scientific fields, it is important to investigate if there are still other groups to be found and whether group membership is consistent over different types of data sets.

Third, what are the binding features of the obtained groups? Groups could be classified based on their formulas but also based on the kind of similarity that they capture. The seminal work of Cronbach and Gleser (1953), for instance, focuses on similarity in the shape of the profiles, in the variance of the scores in the profiles (scatter), and in the means of the scores in the profiles (elevation) and different combinations thereof (Cronbach & Gleser, 1953). Other ways to classify measures might be the kind of association that they capture (e.g., linear, monotone, quadratic) or, more simply, that higher values indicate more or less similar profiles.

Fourth, can we give some recommendations on which measure(s) to use based on the results of the previous steps? Indeed, some groups of measures might be more suited for exploring or answering certain research questions than others.

The remainder of the paper is organized as follows. As we have used empirical data that was previously collected by colleagues, we start with a description of the five data sets used. Then, we explain how we compiled our initial list of similarity measures. Next, we answer the four questions posed above. At the end we discuss some additional conclusions and limitations of this study.

## Data characteristics

In this paper, we will investigate profile similarity as how closely related two sets of ratings on discrete emotion variables are. These ratings can be given by two persons or by aggregating over multiple ratings. To this end, five different empirical data sets have been used: one experience sampling study (ESM), one negative lab interaction with video-mediated recall (VMR), one cross-sectional assessment of a negative lab interaction (Lab) and two cross-cultural data sets with either 14 or 20 measured emotions (Cult14 and Cult20; see Table 1). In the remainder of this paper they are indicated by means of the abbreviations in parentheses. While the variables in all five studies are discrete emotions, the different data sets were selected to differ on multiple characteristics (see Table 1) that we expected to have an influence on the computation of profile similarity values, such as number of emotions, response scale used, intensive longitudinal or cross-sectional design, and inclusion criteria (e.g., romantic partners, students). These differences allow to investigate whether there is an effect of certain data characteristics on the grouping of the measures. We chose to work with empirical rather than simulated data to include a wide array of possible rating patterns, data anomalies, and response tendencies as seen in empirical profiles.

**Table 1:** Five data sets and characteristics.


	PARTICIPANTS	EMOTIONS	SCALE	TIME	EXAMPLE

ESM	94 mixed-gender couples	6	0–100	Longitudinal	(Sels, Ruan, et al., 2020)

VMR	133 mixed-gender couples	12	0–6	Longitudinal	(Boiger et al., 2020)

Lab	101 mixed-gender couples	6	1–7	Cross-sectional	(Sels, Cabrieto, et al., 2020)

Cult14	9300 college students, nested in 48 cultures	14	1–9	Cross-sectional	(Kuppens et al., 2006)

Cult20	1336 participants, nested in 12 cultures	20	1–7	Cross-sectional	(De Roover et al., 2014)


The participants of the ESM study are 94 romantic mixed-gender couples (so, 188 individuals). The study consisted of seven consecutive days of experience sampling. Between six and 14 times a day, depending on whether it was a weekday or the weekend, notifications were sent out for both partners at the same time. At each notification, participants were asked to rate their momentary experience of six emotions (angry, sad, anxious, relaxed, happy, lonely) on a scale from 0 (not at all) to 100 (very). On average, both partners responded to the same notification in 88% of the cases (min = 51%, max = 100%).

The VMR study consisted of a lab interaction where 133 romantic mixed-gender couples from Belgium (n = 57) and Japan (n = 76) discussed a disagreement for 10 minutes in between a neutral and a positive interaction. Afterwards both partners were separated and asked to review a recording of their conversation by means of video-mediated recall. Each 30 seconds the video was paused and the partners rated for themselves how much they had experienced 12 emotions at that time point (annoyed, resigned, hurt, afraid of hurting, guilty, aloof, worried, embarrassed, empathy for my partner, strong, calm, amae) on a scale from 0 (not at all) to 6 (very much).

The lab study consisted again of a negative lab interaction in between the discussion of a neutral and positive topic. 101 mixed-gender couples discussed the most annoying characteristic of their partners for 10 minutes. After the negative conversation, partners were separated to complete a questionnaire on the conversation, rating among others the extent to which they felt six emotions during the interaction (angry, sad, anxious, relaxed, happy, stress) on a scale from 1 (not at all) to 7 (very).

The Cult14 data pertain to a subset of data from the International College Survey 2001. Among 152 questions, 9300 college students from 48 different nations over the world completed 14 emotion ratings. They indicated on a scale from 1 (not at all) to 9 (all the time) how often they felt pleasant, unpleasant, happy, cheerful, sad, angry, proud, grateful, love, guilty, ashamed, worried, stressed and jealous.

The Cult20 study asked 1336 participants from 12 different cultural groups (e.g., first generation Turkish immigrants in Belgium, Latino immigrants in the USA, Flemish, European Americans) to read one to four specific situations (leading to 2714 observations) that differed on valence (positive, negative), social engagement (engaging, disengaging) and social context (friends, home/family, work/school). After reading each situation, the participants indicated to what extent they felt 20 different emotions (upset, irritated, guilty, ashamed, afraid, interested, strong, proud, bored, jealous, ill feelings, close, respect, in debt, relying, resigned, helpful, surprised, worthless, embarrassed) on a scale from 1 (not at all) to 7 (very much).

In the first three datasets, the participants are romantic partners and, thus, two different people. In the last two data sets, the dyads consist of individuals and their cultural norms. The cultural norms are formed by aggregating each emotion variable over individuals per culture. Additionally, we need to account for the normative responses to emotion labels that can create the illusion of similarity (Kenny et al., 2006). In data sets with positive and negative emotions, most profiles will show higher ratings on the positive emotions and lower ratings on the negative emotions. This is a sign of similarity between all people, and not necessarily unique similarity between two people of the same dyad. These normative tendencies can be filtered away by centering, which will then leave us with unique responses (Kenny et al., 2006). In addition, in preliminary work, we have found that computing the Pearson correlation on raw data leads to high similarity values for all dyads and all moments. This unwanted ceiling effect leaves almost no room for differentiating high and low similarity moments and thus complicates studying covariates and, thus, possible causes of high similarity (Carlier et al., 2023). For the longitudinal data, we used person-centering per variable to account for the multilevel structure of the data. Person-centering implies that the average of each individual for each emotion variable was subtracted, which removes both between-variable and between-person differences in means but retains all within-person differences, before computing the similarity between profiles. This way, both universal and person-specific forms of stereotype responding are eliminated. Note that we subtract variable means here and not profile means. We did this separately for men and women, as Kenny et al. (2006) suggest this method for distinguishable dyads. For the lab study we only have one measurement per person making it impossible to person-center data. Therefore, we decided to variable-center each emotion over all men and all women separately. The cultural data sets were cross-sectional, but with multiple measurements per culture. Here, we have chosen to grand-mean center the data to eliminate stereotype responding that was common among all cultures, but to retain the between culture differences in emotion means and thus norms allowing to compute the similarity between individuals and their own cultural norm.

## Compiling a list of similarity measures

To compile a list of potentially useful profile similarity measures, we looked for relevant papers through Google Scholar using combinations of the following keywords: profile, association, agreement, similarity, distance, measure, index and metric. We retained methodological and review papers that discussed similarity and distance measures for all kinds of applications, and involved the comparison of non-binary vectors (Cattell, 1949; Cha, 2007; Cohen, 1969; Deza & Deza, 2009; Furr, 2010; Krebs, 2014; McCrae, 2008; Popescu & Dinu, 2009). Moreover, we searched for R-packages that provide functions to compute similarity, proximity or distance metrics, like proxy, philentropy and base R (Drost, 2018; Meyer & Buchta, 2022; R Core Team, 2022). From these papers and packages we included all measures that have a deterministic way of computing (i.e., they can be computed with closed-form expressions and thus always give the same result, in contrast to for instance stochastic measures) and are preferably symmetrical (i.e., which profile is entered as x- or y-variable does not impact the outcome). We then programmed all 88 selected formulas in R, making use of only the R system library functions such as *sum*(), *mean*(), and *cor*(), with the only exception being *Hmisc::rcorr.cens*() for the Gamma correlation. This allowed us to make sure we could follow the analytical process of each measure and to store all measures in one source file that can easily be shared and adapted for personal use, without having to install a list of varying packages. For an overview of the 88 measures and the developed R syntax, see the Supplementary Material.

## Step 1: Checking uniqueness and applicability of the listed measures

**Figure 1:**
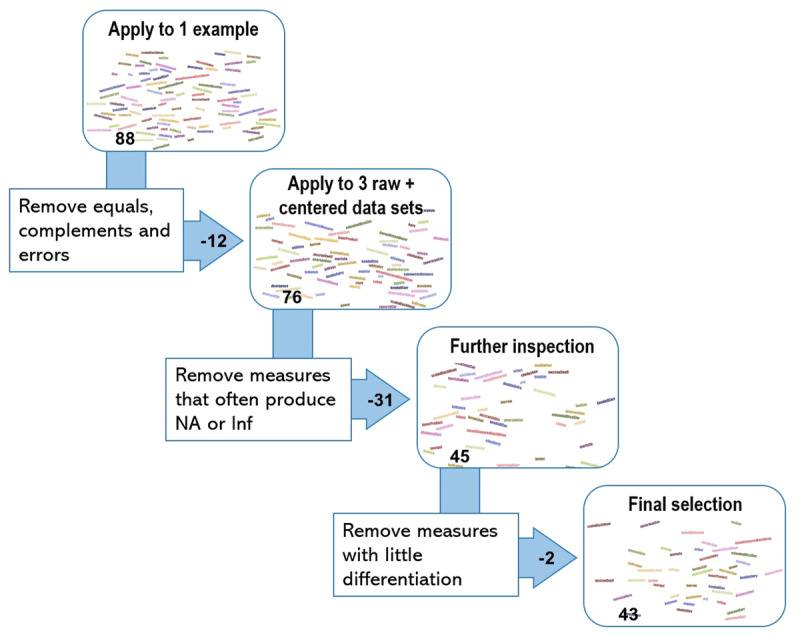
Selection process of 88 to 43 measures.

**Figure 2:**
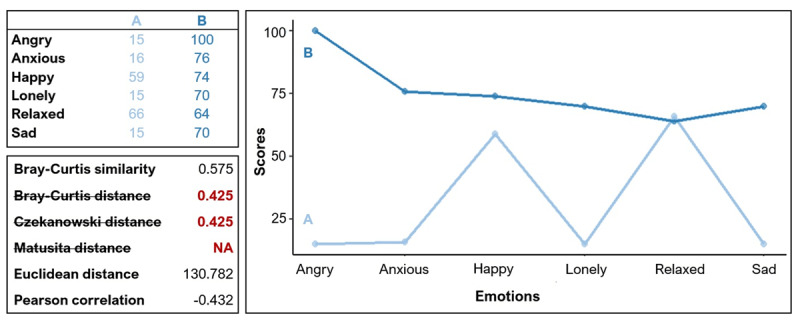
Computation of a few (dis)similarity measures on an example set of profiles. Six measures are indicated among which an erroneous, complementary and duplicate measure.

To check whether the 88 listed measures provide unique information and are applicable for the research problem at hand (see Figure 1), we first applied them to the profiles obtained at the first measurement occasion of the first couple in the first data set, as shown in Figure 2 (top left panel). This real-data example was only used as a first indicator of which measures might be equal or complementary and thus which formulas we had to investigate for this step. We then inspected if any of the selected measures produced duplicate or complementary values. When duplicate values could be brought back to two formula’s being equivalent, only one measure was kept (e.g., Bray-Curtis distance and Czekanowski distance, see Figure 2). If two measures yielded complementary values implying that one quantified similarity and the other dissimilarity (e.g., Bray-Curtis similarity and distance, see Figure 2), only the measure expressing similarity was kept in the selection for sparsity. One measure produced an NA because it could not be computed on values larger than one and was thus removed. As shown in Figure 1, this first inspection phase led to the removal of 12 measures. Note that full information on whether or not a measure was removed and why can be found in the Supplementary Material.

The next phase of the selection process was to apply the remaining measures to three of the five datasets: ESM, VMR, and lab; once using the raw data and once after centering. After selection, we can use the last two data sets as out-of-sample applicability check. As described higher, the ESM and VMR data were person-mean-centered and the lab data were variable-mean-centered. 31 measures that produced at least 5% of NAs or (almost) infinite means for at least two out of the three datasets (ESM, VMR, lab) were excluded. For example, the Canberra distance produced 1659 out of 5150 NAs for the raw ESM data and 2082 out of 5314 NAs on the centered VMR data and was thus excluded at this point. Overall, the measures showed the least NAs on the cross-sectional lab data, indicating that the higher complexity of longitudinal data (e.g., due to more zero ratings, careless responding, outliers) make it harder to compute certain measures. When inspecting the formulas of the discarded measures in these steps, three caveats become apparent: division by 0, log 0 or square root of a negative value. Depending on the formula, the first two will pose a problem when one of the profile items is scored zero, when both partners score the same profile item zero or when an addition or product of the scores on two or more profile items equals zero. Other formulas make use of square roots of single items or additions or products of multiple items. When a bipolar scale around zero is used, or when profile items are centered or standardized, these square roots are more likely to contain negative values, and can thus not be computed. Of course, there are still caveats for the measures that we retained as well. When all profile items are rated the same or rated zero, then there will be a higher likelihood for zero-related issues (e.g., a variance of zero leads to a NA on the Pearson correlation). However, by applying these measures to five different data sets, we have seen that these cases do not happen more than 5% of the time for each data set.

In a final phase, the Ellenberg and Gleason similarities were excluded, because they resulted in a value of 1 for the majority of the observations and thus allow almost no differentiation. Afterwards, we checked whether the resulting list of 43 measures were also applicable to the Cult14 and Cult20 data sets using the same criteria, which was indeed the case.

## Step 2: Clustering the applicable measures

To identify meaningful groups of (dis)similarity measures that yield similar values, we performed a hierarchical cluster analysis using Ward’s criterium on the obtained similarity values per pair of centered profiles for each of the five data sets separately. As mentioned earlier, to account for the presence of normative similarity and possible ceiling effects (Kenny et al., 2006; Carlier et al., 2023), we have centered the emotion variables before applying the similarity measures. This ceiling effect and associated restriction of range may lead to less clear association patterns among the different similarity measures and thus to less stable clustering results. To check whether this pre-processing strategy had the desired effect (i.e., yielding more differentiated and stable clusters), we also applied the hierarchical clustering on similarity values computed on the raw data. The dendrograms obtained for the raw data can be found in the Supplementary material. As expected, there was less agreement on the clusters across raw data sets, indicating that centering indeed gives a more robust clustering result when considering emotions of different valence.

**Figure 3:**
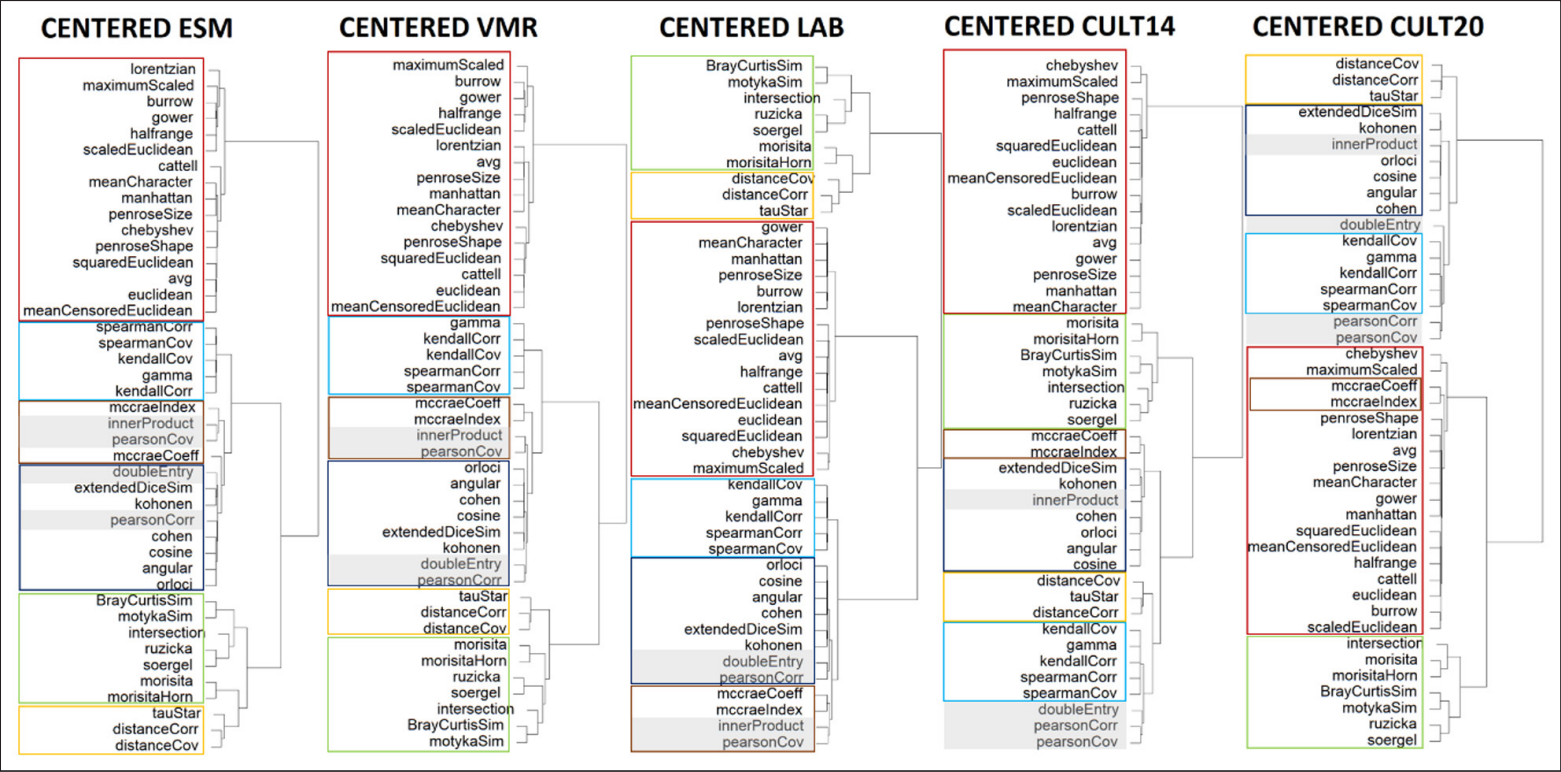
Hierarchical cluster dendrograms on the 5 data sets. Subgroups are indicated: red = Group 1, green = Group 2, yellow = Group 3, blue = Group 4, dark blue = Group 5, and brown = Group 6. Grey indicates inconsistent measures. Abbreviations of all measures can be found in the Supplementary material.

After centering, we computed the association between all profile pairs, using each of the 43 measures per data set, leading to multiple observations per measure. We then computed the distance between each two measures as 1–|*ρ_xy_*| over these observations with *ρ_xy_* denoting the Spearman correlation between a pair of measures. Note that we take the absolute value of the Spearman correlation (Cha, 2007; Cliff et al., 2022) to allow similarity and dissimilarity measures with a strong negative association to be clustered together. The Spearman correlations were multilevel versions, to account for the nesting structure in the intensive longitudinal (occasions nested within persons) and cross-sectional data (participants nested within cultural groups). For this, we have used the *correlation*-package in R, which describes the multilevel correlations as a special case of partial correlation (Makowski et al., 2020). This package runs the correlation test after first having partialized the data based on the (random) effect you choose to adjust for. They define *e_x.z_* as the residuals from the linear prediction of x by z, and then *r_xy.z_* = *r_ex.z,ey.z_*. We obtained dendrograms for the five data sets with *hclust()*, as shown in Figure 3. Cutting the longest distance in the dendrograms indicated the retrieval of mainly three larger clusters for each data set that are built up of six subgroups (see Figure 3 and Table 2). Importantly, these three clusters and subgroups are almost the same across data sets. We will further refer to the general clusters as Cluster 1–3 and to the smaller subgroups as Group 1–6.

**Table 2:** The different obtained clusters and subgroups.


CLUSTER	MEASURES

**Cluster 1: differences**	

Group 1: *difference scores*	Avg(L_1, L_n) distance, Burrows’s Delta, Cattell’s r_p_, Chebyshev’s distance, Euclidean distance, Gower distance, Half-range standardized distance, Lorentzian distance, Manhattan distance, Maximum scaled difference, Mean censored Euclidean distance, Mean character distance, Penrose shape distance, Penrose size distance, Scaled Euclidean distance, Squared Euclidean distance

**Cluster 2: miscellaneous**	

Group 2: *miscellaneous*	Bray-Curtis similarity, Intersection similarity, Morisita’s index of similarity, Morisita-Horn Index of Similarity, Motyka Similarity, Ruzicka similarity, Soergel distance

Group 3: *independence*	Distance correlation, Distance covariance, Tau Star

**Cluster 3: products**	

Group 4: *Rank- and sign-based*	Goodman-Kruskal Gamma correlation, Kendall tau-b rank correlation, Kendall tau-b rank covariance, Spearman rank correlation, Spearman rank covariance

Group 5: *Scaled products*	Angular distance, Cosine similarity, Cohen’s r_c_, Extended Dice similarity, Kohonen similarity, Orloci distance

Group 6: *McCrae’s*	McCrae’s coefficient of profile similarity, McCrae’s index of profile similarity

Inconsistent	Pearson correlation, Double-Entry intraclass correlation, Pearson covariance, Inner product


## Step 3: Interpretation of the clusters

Next, we examined the formulas of the measures that belong to the same cluster and group to identify which elements overlap and are thus important in the way they compute profile similarities. At the level of the three clusters, a clear distinction can be found. The measures in Cluster 1 share pairwise differences at the center of their formulas, and are consequently all unipolar distance measures, with the exception of Cattell’s r_p_. The formula of this measure has similar elements as the Euclidean distance, as is thus a distance in essence, however, due to the specific transformation, Cattell forced this measure to give a bipolar output, similar to a correlation measure. Cluster 3 contains measures with pairwise products at the heart of their formulas. They are all similarity measures, except for the Angular and Orloci distances. They are, however, classified in this cluster because both of these contain the literal Cosine similarity within their formulas, making them strongly related to this measure, albeit in the opposite direction. Cluster 2 is much harder to pinpoint, as it is hard to define a unifying feature and falls somewhere between Cluster 1 and 3. Therefore, we will label it as a miscellaneous category, which is confirmed by the larger distances within this group as seen in the dendrograms (see Figure 3).

**Figure 4:**
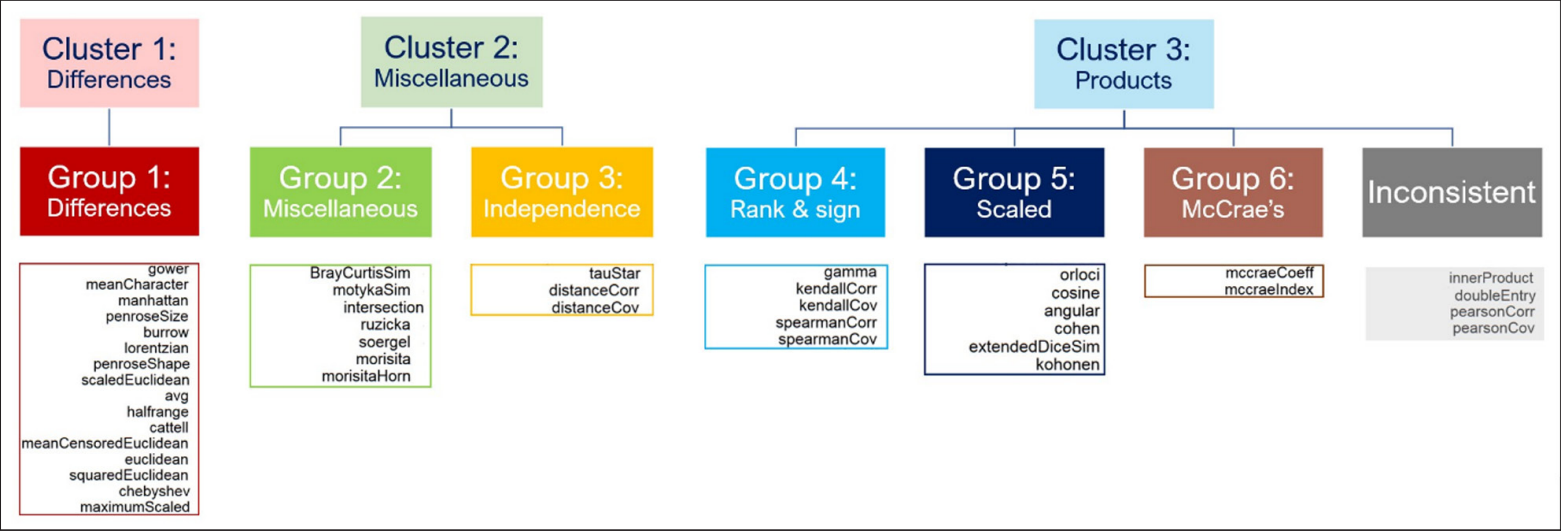
Interpretation of the cluster and subgroup structure of 43 similarity measures.

At the level of the six subgroups that form the building blocks for the three clusters, more nuances can be made. Group 1 equals Cluster 1, and can thus be labeled difference measures. Group 2 is generally defined as the miscellaneous group, since apart from being closer to one another than to the other measures, they have no unique shared features. Five of them contain minima and maxima in their formulas, but the other two do not. Group 3 consists of the measures that capture any form of dependence and in addition, Tau Star is the sign version of the Distance correlation (Bergsma & Dassios, 2014). In three out of five clusterings they form Cluster 2 together with Group 2 and are thus classified therein. But as can be derived from the formula, the Distance correlation and covariance consist of a distance and a correlation part, making them indeed fall somewhere between Cluster 1 and 3. Group 4 consists of the rank- and sign-based correlations and covariances. Group 5 contains a group of other product measures that all have fractions with a pairwise product in the numerator and some sort of scaling in the denominator. In the first three data sets this group also encompasses the Pearson correlation and the Double-Entry intraclass correlation, but not in the last two, making these measures inconsistent. Group 6 consists of McCrae’s coefficient of profile similarity and McCrae’s index of profile similarity. In the first three data sets they also contain the Inner Product and the Pearson covariance, but not in the last two, making also these measures inconsistent. Group 4–6 and the inconsistent measures together form Cluster 3. See Figure 4 for an overview.

As can be seen in Figure 3, one of the best-known and most popular profile similarity measures, the Pearson correlation, cannot exclusively be classified in one subgroup, but rather behaves differently on the data sets. This means that this measure sometimes behaves more like rank-based correlations, then more like scaled products and other times like an entirely different measure. The same is true for two other measures that share almost the same formula: the Pearson covariance and the Double-Entry intraclass correlation. This means that these measures are very sensitive to certain data characteristics, making a consistent interpretation troublesome.

## Step 4: Recommendations

Our last aim was to provide some guidance in helping researchers select measure(s) for their own research. The recommendations depend on whether the researcher has a specific preference for measures with certain characteristics depending on their precise research question or whether they merely want to explore the data at hand without any expectations.

### Starting from specific preferences

When there is a predefined hypothesis, one needs to be aware of the exact question that needs to be answered. The first choice to make, is the one between distance or similarity. When computing a distance, zero indicates perfect similarity and deviations from zero indicate less similarity. When computing a similarity, zero indicates similarity at chance level, while positive and negative deviations from zero indicate more or less similarity than can be expected due to chance. In addition, similarity measures mainly assess whether two profiles look alike, while difference measures assess how close the elements of both profiles actually lie to each other. If the hypothesis can best be answered in terms of a distance, Cluster 1 is the preferred choice. If the hypothesis is expressed in terms of similarity, Cluster 3 is the best choice. If your hypothesis does not really comply with any of the above, Cluster 2 with the independence measures might offer some measures that are worth looking into (see Table 3).

**Table 3:** The different elements in choosing a measure.


CONSIDERATION	RECOMMENDATION

**Direction**	

Distance	Cluster 1

Similarity	Cluster 3

Other/no clear preference	Cluster 2

**Kind of similarity**	

Shape	Group 3 and 4

Shape + scatter + elevation	Group 1 and 5

Non-linear similarity	Group 2, 3 and 4

**Technical**	

Bounded measures	Cluster 1: Cattell’s r_p_,Gower distance Cluster 2: Distance correlation, Group 2 (+) Cluster 3: Cohen, Gamma, Spearman, Kendall, Pearson, and Double-Entry correlations, McCrae’s coefficient of profile agreement, Cosine similarity, Kohonen similarity (+), Extended Dice Similarity (+)

Negative values	Avoid Group 2 and 5


(+): only when computed on positive scores.

**Figure 5:**
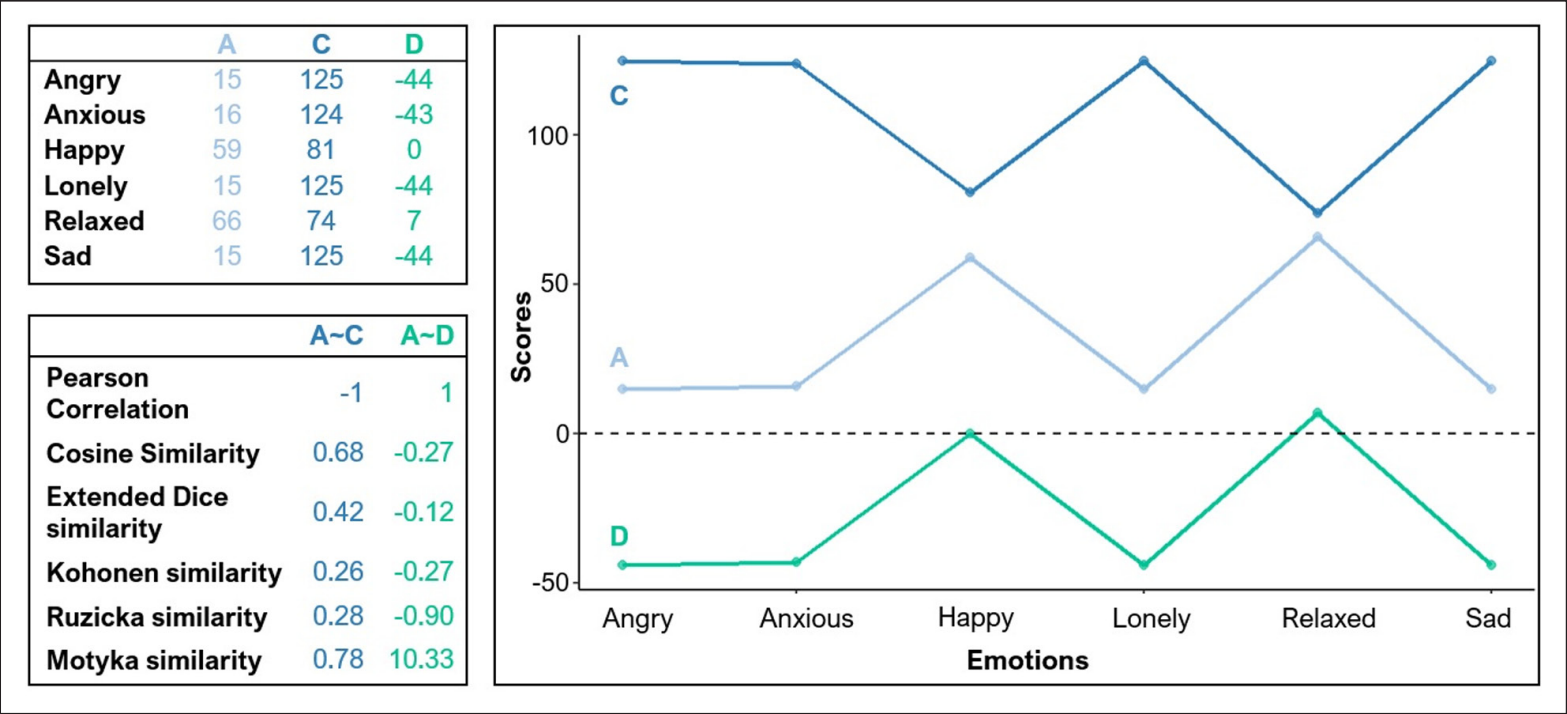
Three profiles with five measures of similarity from Group 2 and 5 and Pearson correlation for reference. All profiles have equal scatters, A and C have an inversed shape, A and D have an equal shape and A is more similar in elevation to D than C.

A second element that influences the choice of measure is whether the hypothesis focuses on shape, scatter and/or elevation similarity. Shape refers to the constellation of the emotion scores in the profiles as indicated by the dots and lines in Figure 5. Some emotion scores are relatively high compared to others in the profiles, implying a particular constellation. Here, this constellation reflects a distinction between positive and negative emotions. Scatter and elevation refer to the variability and central tendency of the profile scores. One could quantify the elevation by computing the mean of the emotion scores in a profile and the scatter by looking at how much the scores deviate from this mean, i.e., their standard deviation. Graphically, it can be seen that profile C in Figure 5 contains higher emotion scores than the other two profiles, while the variation in the individual dots is similar for all three. For a more in depth discussion of these characteristics, see Furr (2010). In other words, is the question related to similarity in the shape of the profiles, to the variance of the scores being the same, to the actual values for each variable lying close to each other in the two profiles or a combination of these (Cronbach & Gleser, 1953; Furr, 2010; Skinner, 1978)? Often shape has been indicated as the most important type of similarity in profile research, however some other important information might be whether people similarly differentiate between emotions (low versus high scatter) and whether one partner has more intense emotions than the other partner (low versus high elevation) (Kenny et al., 2006). When only interested in shape similarity, some subgroups to look at, are Group 3 and Group 4, for the combined shape-scatter-elevation similarity that is Group 1. Group 5 is dominated by shape similarity, but also makes adjustments for scatter and elevation differences. It is of course important to keep in mind that although some measures purely assess the shape of a profile, these three characteristics are statistically intertwined, making it impossible to fully isolate them from each other (Furr, 2010). Apart from these characteristics, one might look for other forms of associations, not per se a linear similarity, then it is worth taking a look at Groups 2, 3 and 4.

A last important consideration in choosing a measure is of a technical nature. Some data characteristics might influence how to interpret your measure. Wide measurement scales and large variances will give even larger outcomes for measures that use squaring like the Euclidean distance-derived measures, or use unscaled products like the covariances and the inner product. When researchers find this harder to interpret, they could also opt for measures bounded between –1 (or 0) and 1. This is the main reason why Cattell (1949) worked out his r_p_, to coerce a distance measure into a correlation format. If this is a requirement then Cattell’s r_p_ and Gower distance are eligible from Cluster 1, the Cohen, Gamma, Pearson, Spearman, Kendall, Distance, and Double-Entry correlations are candidates from Cluster 3 as well as McCrae’s coefficient of profile agreement and Cosine similarity. All measures from the residual cluster are bounded between 0 and 1 when applied to positive values only, same goes for Kohonen and Extended Dice similarity. Also some of the product measures are best interpreted when applied to positive scores only. Multiplying a positive by a negative value leads to negative products and can in turn lead to negative numerators, like in the Kohonen and Extended Dice similarity, but do not necessarily mean inversed relationships, like they do in the correlation measures (see Figure 5). To avoid being inclined to interpret the measures that way, we suggest to avoid Group 2 and 5 when working with negative values.

Lastly, some measures are tailored to specific problems: the McCrae index and coefficient are designed for more extreme profiles like encountered in clinical populations, Cohen’s r_c_ is tailored to scores of which the reverse scores have a meaning as well (e.g. introversion = reversed extraversion) and the Avg(L_1, L_n) distance takes the average of the Manhattan and Chebyshev distances if you have a hard time choosing between these two.

### Sensitivity check

If a researcher is not sure what to expect with respect to the abovementioned considerations, one way to explore similarity in profiles is to choose two very different measures and check whether they yield the same conclusions. One could for instance take the Spearman correlation from the third cluster which is a bipolar and robust similarity measure that is only sensitive to shape, the Mean character distance from the first cluster, which is a unipolar distance measure that is sensitive to scatter, shape and elevation and a measure like Ruzicka similarity from the second cluster, which does something entirely different. Moreover, all are easy to interpret as the former is bounded between [–1,1], the middle is just the average distance between the pairs of scores and the latter goes from 0 to 1 (on positive scores). With respect to the interpretability, it is useful to explore the distribution of the chosen measures when applied to the data. Does the measure display a normal distribution or does it only produce a limited set of values, is it most interpreted at the extremes, does it show outliers? Considering the implications of this distribution can help to choose a measure. For instance, more continuous measures (like the Pearson correlation) are more suited to be linked to continuous covariates.

If after performing this exploration and choosing a set of measures, linking different measures to certain outcome or predictor variables yield the same conclusions about the effect of profile similarity on these variables or vice versa, then the exploration gives you a sound result. If the conclusions change with different measures, one might add nuances like ‘only for shape similarity’ and try to find out why this is the case.

## Discussion

In this paper we have investigated a large body of similarity and distance measures and their applicability in psychological research. We have classified 43 measures into three meaningful clusters that can by further be divided into six subgroups. Based on these classifications we were also able to provide some recommendations. In this discussion we want to draw attention to some extra points.

Of course, still other measures could be useful in psychological research, apart from the 43 we proposed. In the first step we have reduced a list of 88 measures to 43 unique ones. Yet, remember that some of the measures were not excluded because they were erroneous, but because they had almost the same formula as another measure or complemented a similarity measure. Additionally, it is likely that some measures slipped through our search net or will be newly developed over time.

Another point where our recommendations deviate from previous profile and similarity research is in the Pearson correlation. While we see added value in the classical use of a distance measure, we are more hesitant towards the Pearson correlation. A clear advantage is its interpretability between –1 and 1. However, since the Pearson correlation and derivates are very sensitive to certain data artefacts and do not uniquely give similar results as specific other measures on each data set, we would recommend other correlation measures such as the Spearman correlation that are more robust.

A second conclusion we could draw outside of the four posed questions is the necessity of centering when there is a form of common normative similarity that influences the shape of all profiles. For the clustering part, we mentioned that clustering on the raw data yielded varying and unreliable results. Since we are working with emotions, there is a main effect of positive and negative emotions in the raw data. Positive emotions are usually scored higher than negative emotions, which is such a form of normative similarity between all people and not a unique similarity between two profiles. When this main effect is not eliminated by centering, this creates a similar shape for all profiles. When working with only negative emotions or other variables such as personality traits, there might also be a form of common normative similarity, but this does not necessarily influence the shape of all profiles. In this case it is not necessary to center, but rather up to the researcher to decide whether this normative similarity needs to be eliminated before computing the unique similarity between two profiles.

### Limitations and future directions

One limitation of this study is that the results can depend on the empirical data sets used. For the measure review part we have been very strict in selecting the measures that could not be applied under certain data transformations and data sets. We have for instance seen that the two longitudinal data sets yielded more NA or infinite values. This is probably related to the design of the two longitudinal data sets which leads to profiles that contain zero ratings. In the raw data this is a consequence of the measurement scales that included zero. However, even after performing person-mean centering, we observed a lot of profiles that contained at least one zero rating. This was most often the case in the VMR data due to a larger set of emotions and the use of a 0 to 6 Likert scale, which made it more probable that for at least one partner, the intensity of one emotion was never scored larger than zero implying that the person-mean centered scores on that variable also amount to zero. Thus, the selection of measures in this paper is quite strict, but comes with the advantage that the selected measures are likely to return proper values for a large variety of data sets. At the same time, other measures that are left out of the current selection can still be useful on specific, but not all kinds of data (e.g., (non-centered) data that do not contain many zeroes).

This paper has focused on a descriptive explanation of the different measures and their relationships. Inference and reliability based on these measures fall out of the scope of this paper, but are an interesting further step. The choice of measure will also be influenced by a measure’s reliability and some questions are still unsolved, like whether measures computed on only six emotions are less reliable than measures computed on more emotions and if some measures are more robust than others. For future research it would be interesting to assess the reliability and statistical properties of the different measures in a simulation study. This simulation study could for instance focus on the impact of profile and rating characteristics, outliers and correlations between variables that make up a profile. While systematically manipulating shape, scatter and elevation will lead to more insights in whether and how measures capture similarity in these characteristics, altering the measurement scale and number of variables in a profile will tell us more about what kind of data lead to a more reliable assessment of similarity and thus come with more narrow confidence intervals. In addition, some correlation measures assume independence between the (profile) ratings. When working with emotions or other psychological constructs, this assumption is likely violated and it would be worthwhile to investigate the impact of this violation on for instance the width of confidence intervals.

## Conclusion

To conclude with the main recommendations, the Pearson correlation is inconsistent and very sensitive to varying data characteristics. Considering which elements of similarity and profiles are important in a given research question, one might opt for a more robust measure among the many other measures that can be used in psychological research. Additionally, when unwanted sources of common or normative similarity distort the shapes of all profiles in a sample in the same way, person- or variable-centering is recommended to obtain more robust outcomes.

## Data Accessibility Statement

In this study, 5 different data sets have been used that were previously collected by colleagues and were made available to us. These data sets have only been used on a descriptive level and were not used to make statistical inferences. Not all data sets are freely available. The ESM and Lab data sets can be found in the EMOTE database. In the data characteristics section of the manuscript, relevant references to articles based on these and the other data sets have been included. Materials derived from these data are available as supplementary materials on OSF.

## Additional Files

The additional files for this article can be found as follows:

10.5334/pb.1297.s1 S1 Appendix. List of measures. Overview of all measures considered in this paper.

10.5334/pb.1297.s2 S2 Appendix. Step 1 measure reviewing. Overview of the measure reviewing step.

10.5334/pb.1297.s3 S3 Syntax. Similarity functions. R syntax with functions for all considered measures.

10.5334/pb.1297.s4 S4 Figures. Supplementary figures. Dendrograms resulting from hclust().
