# Supplementary material for: A Comparison of Measures for Assessing Profile Similarity in Dyads
Source: Psychol Belg. 2024 Jun 25;64(1):72–84. doi: 10.5334/pb.1297 (PMC11212783; doi:10.5334/pb.1297)
Supplement: S1 Appendix. List of measures. — Overview of all measures considered in this paper. [file pb-64-1-1297-s1.pdf]

Terminology	Color legend
a and b are two score vectors, e.g. a = (1,2,3,4,5) and b = (6,7,8,9,10)	Cluster 1: Differences cluster = (red)
n is the number of elements in the score vectors, needs to be the same for a and b, e.g. n=5	Cluster 2: Miscellaneous cluster = (green)
i indicates the variable in the profile, goes from 1 to n	Cluster 3: Products cluster = (blue)
$\bar{}$ incates the mean being taken, e.g. $\bar{a}$ = the mean of a = 3	

Measure	Formula / Why not selected	Abbreviation
Additive symmetric chi-squared distance	problematic caveat: division by zero	
Angular semi-metric	$\cos^{-1} \frac{\sum_{i=1}^{n} a_i b_i}{\sqrt{\sum_{i=1}^{n} a_i^2} \sqrt{\sum_{i=1}^{n} b_i^2}}$	angular
Avg(L_1, L_n) distance	$\frac{\sum_{i=1}^{n}(\lvert a_i - b_i \rvert) + \max \lvert a_i - b_i \rvert}{2}$	avg
Baroni-Urbani-Buser similarity	problematic caveats: division by zero, square root of negative	
Bhattacharyya distance	problematic caveat: square root of negative	
Bray-Curtis distance	complements similarity	
Bray-Curtis similarity	$\frac{2}{n(\bar{a} + \bar{b})} \sum_{i=1}^{n} \min(a_i, b_i)$	BrayCurtisSim
Burrows' Delta	$\frac{1}{n} \sum_{i=1}^{n} \left\lvert \frac{a_i - b_i}{\sigma_i} \right\rvert$, with σ_i = the sample variance (cross) or dyad variance (longitudinal) in variable i	burrow
Canberra distance	problematic caveat: division by zero	
Canberra metric, Adkins form	problematic caveat: division by zero	
Cattell's r_p	$r_p = \frac{2k - \Sigma(a_i - b_i)^2}{2k + \Sigma(a_i - b_i)^2}$, with k = median $\chi_{df=n-1}$ distribution	cattell

Chebyshev's distance	$\max\lvert a_i - b_i\rvert$	chebyshev
Clark distance	problematic caveat: division by zero	
Cohen's r_c	$\frac{\sum_{i=1}^{n} a_i b_i + nm^2 - m(\sum_{i=1}^{n} a_i + \sum_{i=1}^{n} b_i)}{\sqrt{(\sum_{i=1}^{n} a_i{}^2 + nm^2 - 2m\sum_{i=1}^{n} a_i)(\sum_{i=1}^{n} b_i{}^2 + nm^2 - 2m\sum_{i=1}^{n} b_i)}}$, with $m = \text{midpoint scale}$	cohen
Cosine similarity	$\frac{\sum_{i=1}^{n} a_i b_i}{\sqrt{\sum_{i=1}^{n} a_i{}^2}\sqrt{\sum_{i=1}^{n} b_i{}^2}}$	cosine
Czekanowski distance/similarity	same as Bray-Curtis	
Dice distance	complements similarity	
Distance correlation	$\sqrt{\frac{\frac{1}{n^2}\sum_{i=1}^{n}\sum_{j=1}^{n} D(a_i,a_j)D(b_i,b_j)}{\sqrt{\frac{1}{n^2}\sum_{i=1}^{n}\sum_{j=1}^{n} D(a_i,a_j)^2 * \frac{1}{n^2}\sum_{i=1}^{n}\sum_{j=1}^{n} D(b_i,b_j)^2}}}$ with D() all pairwise double centered Euclidean distances	distanceCorr
Distance covariance	$\sqrt{\frac{1}{n^2}\sum_{i=1}^{n}\sum_{j=1}^{n} D(a_i,a_j)D(b_i,b_j)}$ with D() all pairwise double centered Euclidean distances	distanceCov
Divergence Chi-Squared	problematic caveat: division by zero	
Double-entry ICC	$\frac{\sum_{i=1}^{n}(\text{append}(a,b)_i - \overline{\text{append}(a,b)})(\text{append}(b,a)_i - \overline{\text{append}(b,a)})}{\sqrt{\sum_{i=1}^{n}(\text{append}(a,b)_i - \overline{\text{append}(a,b)})^2}\sqrt{\sum_{i=1}^{n}(\text{append}(b,a)_i - \overline{\text{append}(b,a)})^2}}$, with append(a,b) = adding values of profile b behind values of profile a	doubleEntry
Ellenberg similarity	>75% of values = 1, little differentiation possible	
Euclidean distance	$\sqrt{\sum_{i=1}^{n}(a_i - b_i)^2}$	euclidean
Extended dice similarity	$\frac{2\sum_{i=1}^{n} a_i b_i}{\sum_{i=1}^{n} a_i{}^2 + \sum_{i=1}^{n} b_i{}^2}$	extendedDiceSim

Fidelity similarity	problematic caveat: square root of negative	
Gamma correlation (Goodman-Kruskal)	$\frac{\sum_{i<j}^{n} a_{ij} b_{ij}}{n}$, with $a_{ij} = \begin{cases} 1 \text{ if } a_i \leq a_j \\ -1 \text{ if } a_i > a_j \end{cases}$ and $b_{ij} = \begin{cases} 1 \text{ if } b_i \leq b_j \\ -1 \text{ if } b_i > b_j \end{cases}$	gamma
Gleason similarity	>75% of values = 1, little differentiation possible	
Gower distance	$\frac{1}{n} \sum_{i=1}^{n} \frac{\lvert a_i - b_i \rvert}{range_i}$, with $range_i$ = max value - min value of variable i in sample (cross) or dyad (longitudinal)	gower
Half-range standardized distance	$\sqrt{\sum_{i=1}^{n} \left(\frac{a_i - b_i}{HR_i} \right)^2}$, with HR_i = half-range$_i = \frac{range_i}{2}$	halfrange
Harmonic mean similarity	problematic caveat: division by zero	
Hellinger distance	problematic caveat: square root of negative	
Horn's Index of similarity	problematic caveat: logarithm of zero	
Inner product similarity	$\sum_{i=1}^{n} (a_i b_i)^2$	innerProduct
Intersection similarity	$\sum_{i=1}^{n} \min(a_i, b_i)$	intersection
Jaccard similarity	same as Kohonen	
Jeffrey's distance	problematic caveat: logarithm of zero, division by zero	
Jensen difference	problematic caveat: logarithm of zero	
Jensen-Shannon distance	same as Jensen difference	
K Divergence	problematic caveat: logarithm of zero, division by zero	

Kendall's tau-b rank correlation	$\frac{\sum_{i,j=1}^{n} a_{ij} b_{ij}}{\sqrt{\sum_{i,j=1}^{n} a_{ij}^{2}}\sqrt{\sum_{i,j=1}^{n} b_{ij}^{2}}}$, with $a_{ij} = \begin{cases} 1 \text{ if } a_i < a_j \\ 0 \text{ if } a_i = a_j \\ -1 \text{ if } a_i > a_j \end{cases}$ and $b_{ij} = \begin{cases} 1 \text{ if } b_i < b_j \\ 0 \text{ if } b_i = b_j \\ -1 \text{ if } b_i > b_j \end{cases}$	kendallCor
Kendall's tau-b rank covariance	$\sum_{i,j=1}^{n} a_{ij} b_{ij}$, with $a_{ij} = \begin{cases} 1 \text{ if } a_i < a_j \\ 0 \text{ if } a_i = a_j \\ -1 \text{ if } a_i > a_j \end{cases}$ and $b_{ij} = \begin{cases} 1 \text{ if } b_i < b_j \\ 0 \text{ if } b_i = b_j \\ -1 \text{ if } b_i > b_j \end{cases}$	kendallCov
Kohonen similarity	$\frac{\sum_{i=1}^{n} a_i b_i}{\sum_{i=1}^{n} a_i b_i + \sum_{i=1}^{n} (a_i - b_i)^2}$	kohonen
Kulczynski similarity 1	problematic caveat: division by zero	
Kulczynski similarity 2	problematic caveat: division by zero	
Kullback-Leibler	problematic caveat: logarithm of zero, division by zero	
Kumar-Hassebrook (PCE) similarity	same as Kohonen	
Kumar-Johnson	problematic caveat: square root of negative, division by zero	
Lorentzian distance	$\sum_{i=1}^{n} \ln(1 + \lvert a_i - b_i \rvert)$	lorentzian
Manhattan distance	$\sum_{i=1}^{n} \lvert a_i - b_i \rvert$	manhattan
Matusita distance	problematic caveat: square root of negative	
Maximum scaled difference	$\max\left(\frac{(a_i - b_i)^2}{\sigma_i^{2}}\right)$, with σ_i =the sample variance (cross) or dyad variance (longitudinal) in variable i	maximumScaled
McCrae's coefficient of profile agreement	$\frac{I_{pa}}{\sqrt{(n-2) + I_{pa}^{2}}}$, with $I_{pa} = \frac{n + 2\sum_{i=1}^{n}\left(\frac{a_i + b_i}{2}\right)^2 - \sum_{i=1}^{n}(a_i - b_i)^2}{\sqrt{10n}}$	mccraeCoeff

McCrae's index of profile agreement	$I_{pa} = \frac{n + 2\sum_{i=1}^{n}\left(\frac{a_i + b_i}{2}\right)^2 - \sum_{i=1}^{n}(a_i - b_i)^2}{\sqrt{10n}}$	mccraeIndex
Mean censored Euclidean distance	$\sqrt{\frac{\sum_{i=1}^{n}(a_i - b_i)^2}{\sum_{i=1}^{n} 1_{a_i{}^2+b_i{}^2\neq 0}}}$	meanCensoredEuclidean
Mean character distance	$\frac{1}{n}\sum_{i=1}^{n}\lvert a_i - b_i\rvert$	meanCharacter
Morisita's index of similarity	$\frac{2\sum_{i=1}^{n} a_i b_i}{(\lambda_a + \lambda_b)\sum_{i=1}^{n} a_i \sum_{i=1}^{n} b_i}$, with $\lambda_a = \frac{\sum_{i=1}^{n} a_i (a_i - 1)}{\sum_{i=1}^{n} a_i \left(\sum_{i=1}^{n} a_i - 1\right)}$ and $\lambda_b = \frac{\sum_{i=1}^{n} b_i (b_i - 1)}{\sum_{i=1}^{n} b_i \left(\sum_{i=1}^{n} b_i - 1\right)}$	morisita
Morisita-Horn Index of Similarity	$\frac{2\sum_{i=1}^{n} a_i b_i}{\sum_{i=1}^{n} a_i{}^2 \frac{\bar{b}}{\bar{a}} + \sum_{i=1}^{n} b_i{}^2 \frac{\bar{a}}{\bar{b}}}$	morisitaHorn
Motyka distance	complements similarity	
Motyka similarity	$\frac{\sum_{i=1}^{n} \min(a_i, b_i)}{\sum_{i=1}^{n}(a_i + b_i)}$	motykaSim
Neyman chi-squared distance	problematic caveat: division by zero	
Non-intersection distance	complements intersection	
Orloci distance	$\sqrt{2\left(1 - \frac{\sum_{i=1}^{n} a_i b_i}{\sqrt{\sum_{i=1}^{n} a_i{}^2}\sqrt{\sum_{i=1}^{n} b_i{}^2}}\right)}$	orloci
Pearson chi-squared distance	problematic caveat: division by zero	
Pearson correlation	$\frac{\sum_{i=1}^{n}(a_i - \bar{a})(b_i - \bar{b})}{\sqrt{\sum_{i=1}^{n}(a_i - \bar{a})^2}\sqrt{\sum_{i=1}^{n}(b_i - \bar{b})^2}}$	pearsonCorr
Pearson covariance	$\frac{\sum_{i=1}^{n}(a_i - \bar{a})(b_i - \bar{b})}{n - 1}$	pearsonCov

Penrose shape distance	$\sqrt{\sum_{i=1}^{n}\left((a_i-\bar{a})-(b_i-\bar{b})\right)^2}$	penroseShape
Penrose size distance	$\sqrt{n}\sum_{i=1}^{n}\lvert a_i-b_i\rvert$	penroseSize
Precision	problematic caveat: division by zero	
Probabilistic symmetric chi-squared distance	problematic caveat: division by zero	
Roberts similarity	problematic caveat: division by zero	
Ruzicka similarity	$\frac{\sum_{i=1}^{n}\min(a_i,b_i)}{\sum_{i=1}^{n}\max(a_i,b_i)}$	ruzicka
Scaled Euclidean distance	$\sqrt{\sum_{i=1}^{n}\frac{(a_i\;-\;b_i)^2}{\sigma_i{}^2}}$, with σ_i =the sample variance (cross) or dyad variance (longitudinal) in variable i	scaledEuclidean
Soergel distance	$\frac{\sum_{i=1}^{n}\lvert a_i-b_i\rvert}{\sum_{i=1}^{n}\max(a_i,b_i)}$	soergel
Sørensen distance	same as Bray-Curtis	
Spearman correlation	$\frac{\sum_{i=1}^{n}\left(R(a_i)-\overline{R(a)}\right)\left(R(b_i)-\overline{R(b)}\right)}{\sqrt{\sum_{i=1}^{n}\left(R(a_i)-\overline{R(a)}\right)^2}\sqrt{\sum_{i=1}^{n}\left(R(b_i)-\overline{R(b)}\right)^2}}$, with R() = the rank(s) of the profile item(s)	spearmanCorr
Spearman covariance	$\frac{\sum_{i=1}^{n}\left(R(a_i)-\overline{R(a)}\right)\left(R(b_i)-\overline{R(b)}\right)}{n\;-\;1}$, with R() = the rank(s) of the profile item(s)	spearmanCov
Squared chi-squared distance	problematic caveat: division by zero	
Squared-Chord distance	problematic caveat: square root of negative	
Squared Euclidean Distance	$\sum_{i=1}^{n}(a_i\;-\;b_i)^2$	squaredEuclidean
Statisitcal or Standardized distance	same as scaled euclidean	

Symmetric χ2-distance	problematic caveat: division by zero, square root of negative	
Symmetric χ2-measure	problematic caveat: division by zero	
Taneja distance	problematic caveat: logarithm of zero, square root of negative	
Tanimoto distance	same as Soergel	
Tau Star	$\frac{1}{n^4}\sum_{i,j,k,l=1}^{n} \mathrm{sign}(\lvert a_i - a_j\rvert + \lvert a_k - a_l\rvert - \lvert a_i - a_k\rvert - \lvert a_j - a_l\rvert) * \mathrm{sign}(\lvert b_i - b_j\rvert + \lvert b_k - b_l\rvert - \lvert b_i - b_k\rvert - \lvert b_j - b_l\rvert)$	tauStar
Topsøe distance	problematic caveat: logarithm of zero, division by zero	
Wave-Hedges distance	problematic caveat: division by zero	
Whittaker index of association	problematic caveat: division by zero	

Reference list

Baroni-Urbani, C., & Buser, M. W. (1976). Similarity of Binary Data. Systematic Biology, 25(3), 251–259. https://doi.org/10.2307/2412493

Bergsma, W., & Dassios, A. (2014). A consistent test of independence based on a sign covariance related to Kendall’s tau. Bernoulli, 20(2). https://doi.org/10.3150/13-BEJ514

Bhattacharyya, A. (1946). On a Measure of Divergence between Two Multinomial Populations. Sankhyā: The Indian Journal of Statistics (1933-1960), 7(4), 401–406.

Bravais, A. (1844). Analyse mathématique sur les probabilités des erreurs de situation d’un point. Impr. Royale. http://www.mdz-nbn-resolving.de/urn/resolver.pl?urn=urn:nbn:de:bvb:12-bsb10053322-5

Bray, J. R., & Curtis, J. T. (1957). An Ordination of the Upland Forest Communities of Southern Wisconsin. Ecological Monographs, 27(4), 326–349. https://doi.org/10.2307/1942268

Burrows, J. (2002). ‘Delta’: A Measure of Stylistic Difference and a Guide to Likely Authorship. Literary and Linguistic Computing, 17(3), 267–287. https://doi.org/10.1093/llc/17.3.267

Cattell, R. B. (1949). Rpand other coefficients of pattern similarity. Psychometrika, 14(4), 279–298. https://doi.org/10.1007/BF02289193

Cha, S.-H. (2007). Comprehensive Survey on Distance/Similarity Measures between Probability Density Functions. International Journal of Mathematical Models and Methods in Applied Sciences, 1(4), Article 4.

Clairaut, A. (1741). Éléments de Géométrie. Lambert et Durand.

Clark, P. J. (1952). An Extension of the Coefficient of Divergence for Use with Multiple Characters. Copeia, 1952(2), 61–64. https://doi.org/10.2307/1438532

Cohen, J. (1969). rc: A profile similarity coefficient invariant over variable reflection. Psychological Bulletin, 71(4), 281–284. https://doi.org/10.1037/h0026865

Czekanowski, J. (1909). Zur differential Diagnose der Neandertalgruppe. Korrespondenz-Blatt Der Deutschen Gesellschaft Fur Anthropologie, 40, 44–47.

Deza, E., & Deza, M. M. (2009). Encyclopedia of Distances. Springer Berlin Heidelberg. https://doi.org/10.1007/978-3-642-00234-2

Dice, L. R. (1945). Measures of the Amount of Ecologic Association Between Species. Ecology, 26(3), 297–302. https://doi.org/10.2307/1932409

Dodge, Y. (Ed.). (2006). The Oxford dictionary of statistical terms (First published in paperback 2006). Oxford University Press.

Ellenberg, H. (1956). Aufgaben und Methoden der Vegetationskunde. Einfuhrung in Die Phytologie, 4. https://cir.nii.ac.jp/crid/1573668924482426112

Gleason, H. A. (1920). Some Applications of the Quadrat Method. Bulletin of the Torrey Botanical Club, 47(1), 21–33. https://doi.org/10.2307/2480223

Goodman, L. A., & Kruskal, W. H. (1954). Measures of association for cross classifications. Journal of the American Statistical Association, 49, 732–769. https://doi.org/10.2307/2281536

Gower, J. C. (1971). A General Coefficient of Similarity and Some of Its Properties. Biometrics, 27(4), 857–871. https://doi.org/10.2307/2528823

Hedges, T. (1976). An empirical modification to linear wave theory. Ice Proceedings, 61, 575–579. https://doi.org/10.1680/iicep.1976.3408

Heiser, W. (2011, May 31). Clustering, Part 1: Proximity Measures & Hierarchical Clustering [Lecture].

Hellinger, E. (1909). Neue Begründung der Theorie quadratischer Formen von unendlichvielen Veränderlichen. Journal für die reine und angewandte Mathematik, 1909(136), 210–271. https://doi.org/10.1515/crll.1909.136.210

Horn, H. S. (1966). Measurement of “Overlap” in Comparative Ecological Studies. The American Naturalist, 100(914), 419–424. https://doi.org/10.1086/282436

Jaccard, P. (1901). Étude comparative de la distribution florale dans une portion des Alpes et des Jura. Bulletin Del La Société Vaudoise Des Sciences Naturelles, 37, 547–579.

Jeffreys, H. (1946). An invariant form for the prior probability in estimation problems. Proceedings of the Royal Society of London. Series A. Mathematical and Physical Sciences, 186(1007), 453–461. https://doi.org/10.1098/rspa.1946.0056

Kendall, M. G. (1938). A New Measure Of Rank Correlation. Biometrika, 30(1–2), 81–93. https://doi.org/10.1093/biomet/30.1-2.81

Kulczyński, S. (1928). Die pflanzenassoziationen der pieninen (Vol. 3). Imprimerie de l’Université.

Kullback, S., & Leibler, R. A. (1951). On Information and Sufficiency. The Annals of Mathematical Statistics, 22(1), 79–86.

Kumar, B. V. K. V., & Hassebrook, L. (1990). Performance measures for correlation filters. Appl. Opt., 29(20), 2997–3006. https://doi.org/10.1364/AO.29.002997

Kumar, P., & Johnson, A. (2005). On A Symmetric Divergence Measure And Information Inequalities.

Lance, G., & Williams, W. T. (1967). Mixed-Data Classificatory Programs I - Agglomerative Systems. Aust. Comput. J. https://www.semanticscholar.org/paper/Mixed-Data-Classificatory-Programs-I-Agglomerative-Lance-Williams/4072b6353dd761ba0c219a4b860073f08bb3db27

Lin, J. (1991). Divergence measures based on the Shannon entropy. IEEE Transactions on Information Theory, 37(1), 145–151. https://doi.org/10.1109/18.61115

Matusita, K. (1955). Decision Rules, Based on the Distance, for Problems of Fit, Two Samples, and Estimation. The Annals of Mathematical Statistics, 26(4), 631–640.

Maxwell, B. A., & Buddemeier, R. W. (2002). Coastal typology development with heterogeneous data sets. Regional Environmental Change, 3(1), 77–87. https://doi.org/10.1007/s10113-001-0034-8

McCrae, R. R. (1993). Agreement of Personality Profiles Across Observers. Multivariate Behavioral Research, 28(1), 25–40. https://doi.org/10.1207/s15327906mbr2801_2

Meyer, D., & Buchta, C. (2022). Package ‘proxy.’

Morisita, M. (1959). Measuring of interspecific association and similarity between assemblages. Mem Fac Sci Kyushu Univ Ser E Biol, 3, 65–80.

Motyka, J. (1947). O zadaniach i metodach badań geobotanicznych: Sur les buts et les méthodes des recherches géobotaniques. Nakładem Uniwersytetu Marii Curie-Skłodowkiej.

Neyman, J. (1949). Contribution to the Theory of the {χ superscript 2} Test. In Proceedings of the [First] Berkeley Symposium on Mathematical Statistics and Probability (Vol. 1, pp. 239–274). University of California Press. https://projecteuclid.org/ebooks/berkeley-symposium-on-mathematical-statistics-and-probability/Proceedings-of-the-First-Berkeley-Symposium-on-Mathematical-Statistics-and/chapter/Contribution-to-the-Theory-of-the-%cf%87-superscript-2-Test/bsmsp/1166219208

Orloci, L. (1967). An Agglomerative Method for Classification of Plant Communities. Journal of Ecology, 55(1), 193–206. https://doi.org/10.2307/2257725

Pearson, K. (1895). Note on Regression and Inheritance in the Case of Two Parents. Proceedings of the Royal Society of London Series I, 58, 240–242.

Pearson, K. (1900). X. On the criterion that a given system of deviations from the probable in the case of a correlated system of variables is such that it can be reasonably supposed to have arisen from random sampling. The London, Edinburgh, and Dublin Philosophical Magazine and Journal of Science, 50(302), 157–175. https://doi.org/10.1080/14786440009463897

Penrose, L. S. (1952). Distance, Size and Shape. Annals of Eugenics, 17(1), 337–343. https://doi.org/10.1111/j.1469-1809.1952.tb02527.x

Roberts, D. W. (1986). Ordination on the Basis of Fuzzy Set Theory. Vegetatio, 66(3), 123–131.

Robinson, W. S. (1957). The Statistical Measurement of Agreement. American Sociological Review, 22(1), 17–25. https://doi.org/10.2307/2088760

Ružička, M. (1958). Anwendung mathematisch-statisticher Methoden in der Geobotanik (synthetische Bearbeitung von Aufnahmen). Biologia, Bratislava, 13, 647.

Sibson, R. (1969). Information radius. Zeitschrift Für Wahrscheinlichkeitstheorie Und Verwandte Gebiete, 14(2), 149–160. https://doi.org/10.1007/BF00537520

Sørensen, T. (1948). A Method of Establishing Groups of Equal Amplitude in Plant Sociology Based on Similarity of Species Content and Its Application to Analyses of the Vegetation on Danish Commons. Munksgaard in Komm.

Spearman, C. (1904). The Proof and Measurement of Association between Two Things. The American Journal of Psychology, 15(1), 72–101. https://doi.org/10.2307/1412159

Székely, G. J., Rizzo, M. L., & Bakirov, N. K. (2007). Measuring and testing dependence by correlation of distances. The Annals of Statistics, 35(6), 2769–2794. https://doi.org/10.1214/009053607000000505

Taneja, I. J. (1995). New Developments in Generalized Information Measures. In P. W. Hawkes (Ed.), Advances in Imaging and Electron Physics (Vol. 91, pp. 37–135). Elsevier. https://doi.org/10.1016/S1076-5670(08)70106-X

Tanimoto, T. T. (1958). An Elementary Mathematical theory of Classification and Prediction. IBM.

Topsoe, F. (2000). Some inequalities for information divergence and related measures of discrimination. IEEE Transactions on Information Theory, 46(4), 1602–1609. https://doi.org/10.1109/18.850703

Whittaker, R. H. (1952). A Study of Summer Foliage Insect Communities in the Great Smoky Mountains. Ecological Monographs, 22(1), 2–44. https://doi.org/10.2307/1948527

Zadeh, L. A. (1965). Fuzzy sets. Information and Control, 8(3), 338–353. https://doi.org/10.1016/S0019-9958(65)90241-X
