# Supplementary material for: A Comparison of Measures for Assessing Profile Similarity in Dyads
Source: Psychol Belg. 2024 Jun 25;64(1):72–84. doi: 10.5334/pb.1297 (PMC11212783; doi:10.5334/pb.1297)

**Dendrogram plots**

RAW CULT20

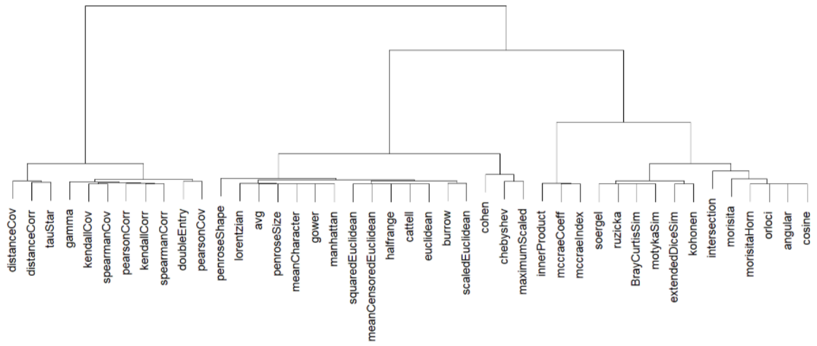


RAW CULT14

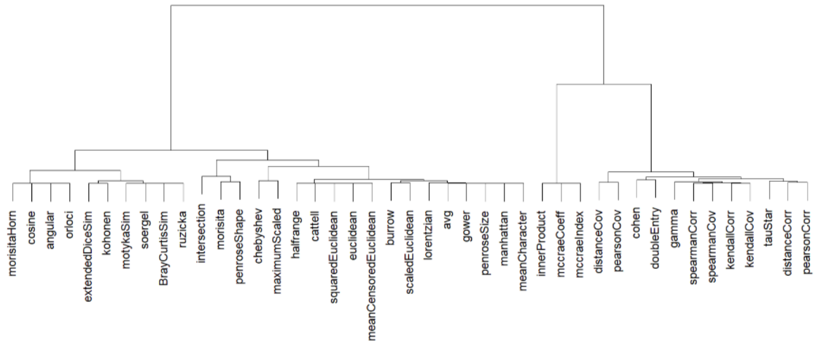


RAW LAB

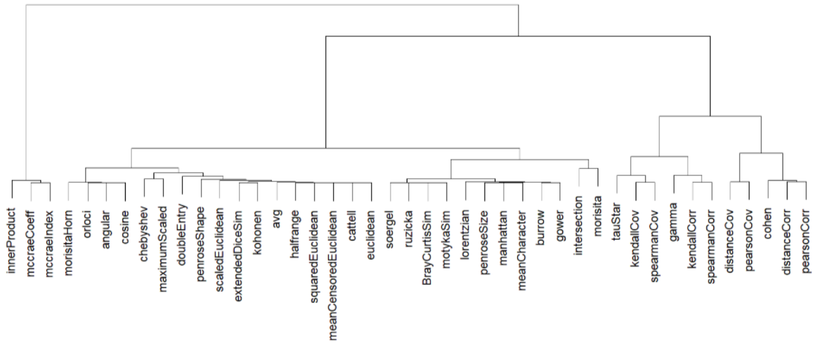


RAW VMR

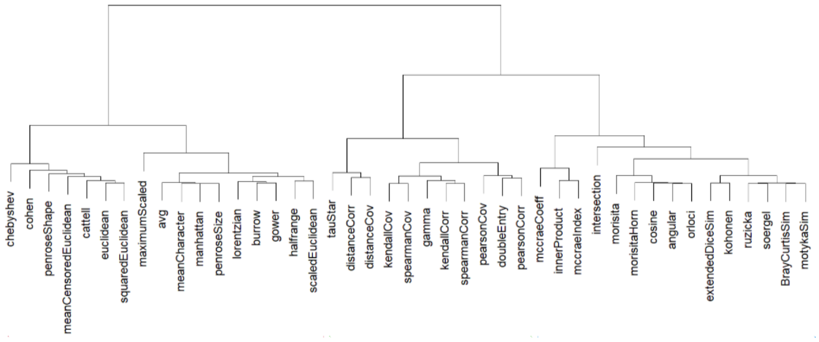


RAW ESM

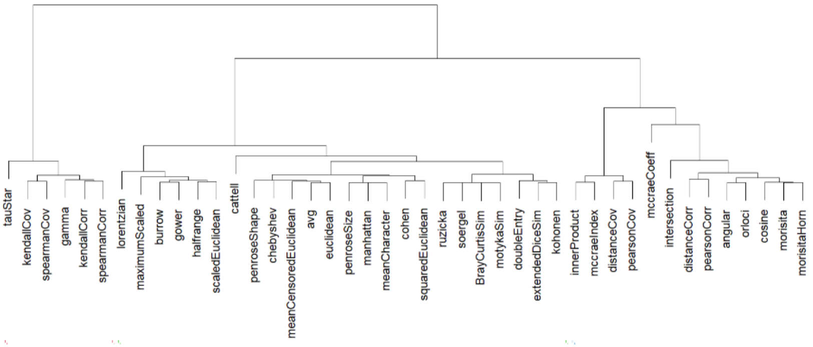

## CENTERED ESM

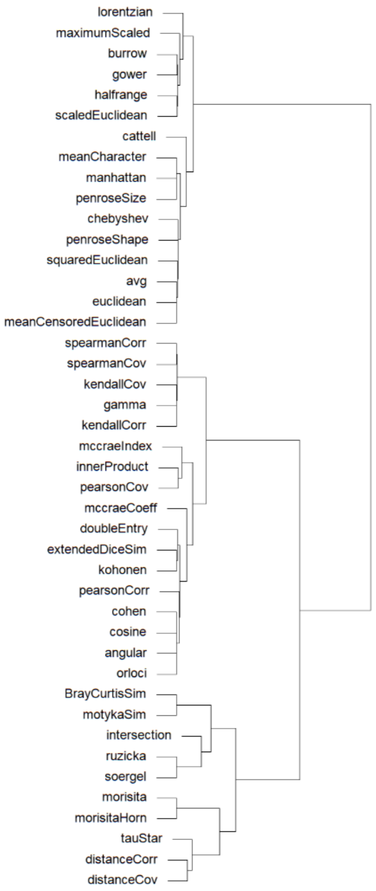


## CENTERED VMR

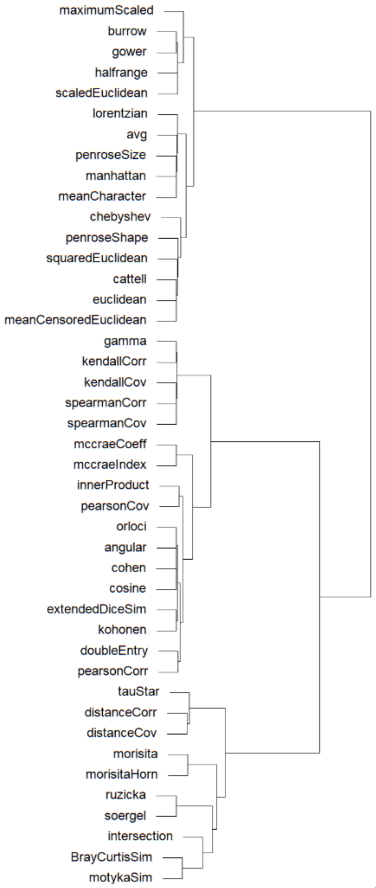


## CENTERED LAB

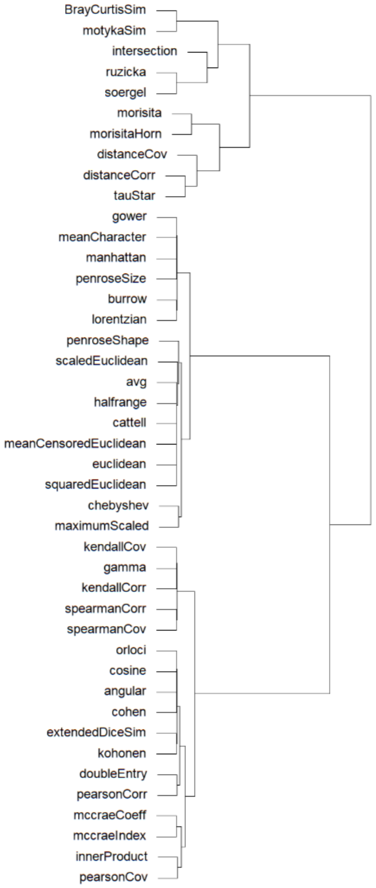


## CENTERED CULT14

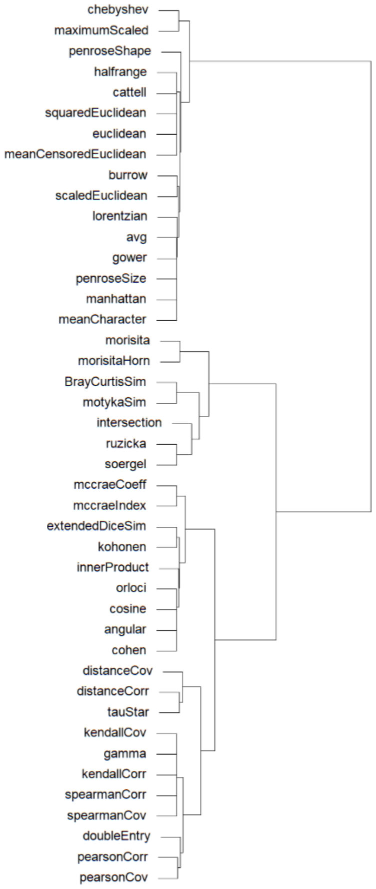


## CENTERED CULT20

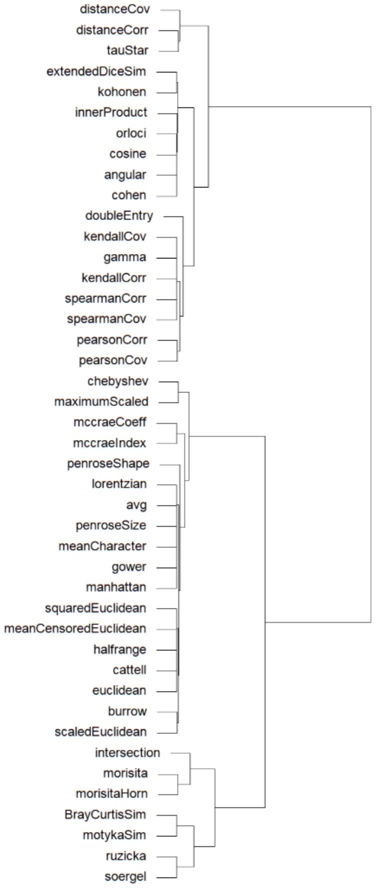

Supplement: S4 Figures. Supplementary figures. — Dendrograms resulting from hclust(). [file pb-64-1-1297-s4.pdf]
